# Unveiling the Journey to Community Reintegration: Results from a Qualitative Study with Sexual Offenders on Parole

**DOI:** 10.1177/0306624X251324661

**Published:** 2025-02-27

**Authors:** Ana Rita Cardoso, Jorge Quintas, Gilda Santos

**Affiliations:** 1 University of Porto, Portugal; 2 CIJ – Centre for Interdisciplinary Research on Justice, Porto, Portugal

**Keywords:** sex offenders, community reintegration, reintegration barriers, parole, sex offender registry

## Abstract

After serving a prison sentence, sex offenders embark on a community reintegration process, where they usually face several barriers that might negatively affect their successful return to society. Results from semi-structured interviews conducted with sex offenders revealed that participants perceive the economic difficulties, the nature of the crime, and the stigma as the main social factors hindering their community reintegration process. Additionally, prison and parole support are described as insufficient, lacking focus on the specific needs of the participants to act as an effective help. On the contrary, housing and affective relationships were not perceived as major obstacles to a successful reintegration. These findings highlight the need for policies that promote economic opportunities, reduce stigma, and enhance both prison-based and post-release support to facilitate a more effective reintegration process.

## Introduction

Public and governmental responses to sexual offenders are often shaped by the interaction between media narratives and public opinion. A dynamic of fear has led to the implementation of measures aimed at reducing risks posed by offenders living in communities, such as registration and notification laws, as well as residency restrictions (Brown et al., 2007; Schiavone & Jeglic, 2009). Within this broader context, the Portuguese framework for reintegrating sexual offenders presents unique challenges, influenced by distinct legal and cultural factors. Unlike Anglo-Saxon countries, such as the United States, where reintegration policies prioritize punitive measures and extensive monitoring (McAlinden, 2006; Robbers, 2009), Portugal may demonstrate greater flexibility in balancing public safety with offender rehabilitation.

In the U.S., Megan’s Law mandates that all states establish community notification systems for registered sexual offenders, ensuring public access to their information (Harris & Socia, 2016; Levenson & Cotter, 2005b; Schiavone & Jeglic, 2009). On the contrary, despite some appeals from far-right parties, so far, Portugal does not have public registration or community notification laws for sex offenders. Instead, information on sex offenders is stored in a restricted database accessible only to judicial and law enforcement authorities (as previewed in Law no. 103/2015, of August 24; Diário da República, 2015), reflecting the country’s focus on offender rehabilitation and society reintegration, which includes sex offenders. This aligns with the Portuguese commitment to privacy rights and balancing public safety with the opportunity for offenders to reintegrate successfully (Gomes et al., 2018).

Sex offenders are widely regarded as one of the most stigmatized groups of criminals, facing severe consequences such as social ostracism, public recognition, and exclusion from social groups, organizations, and activities (Mingus & Burchfield, 2012; Tewksbury & Lees, 2006). Registration and notification laws, like Megan’s Law, exacerbate this stigma by publicly labelling the individuals as sexual offenders. Therefore, this legal and social branding often prevents them from re-establishing any positive standing within society post-incarceration, as the label of sex offender becomes their primary status (Robbers, 2009). In addition to these challenges, previous studies have demonstrated that sexual offenders face the most significant barriers to reintegration, such as difficulties in rebuilding relationships with family, friends, and neighbors, securing stable housing and employment, complying with legal and civic restrictions, and overcoming the pervasive stigma of being a former incarcerated individual (Brown et al., 2007; Visher & Travis, 2003). They are also often met with more severe resistance from the community, including harassment and threats, and may endure stricter parole and probation conditions, such as electronic monitoring (Burchfield & Mingus, 2008; Lussier et al., 2011). Thus, these major barriers to reintegration—such as securing employment, stable housing, rebuilding personal relationships, and managing the societal stigma—further difficult the process for sexual offenders striving to re-enter the community (Robbers, 2009; Tewksbury & Lees, 2006).

Employment is seen as a crucial pillar for successful reintegration, helping former offenders develop new roles, skills, and work experience while promoting a normative routine and pro-social connections (Solomon et al., 2004). However, employment opportunities for sex offenders are heavily restricted in countries with stringent punitive measures, particularly for positions involving contact with vulnerable populations, such as children (Rydberg, 2018). For example, the United States Adam Walsh Child Protection and Safety Act (2006) prohibits offenders from working in environments like schools, community centers, or day-care facilities. Furthermore, background checks have become more accessible, often disqualifying offenders from jobs in fields such as private security or healthcare (Stoll & Bushway, 2008). While sex offenders in Portugal may face employment challenges, direct occupational bans are less common, with limitations generally focusing on preventing contact with children, when imposed by the court (Portuguese Penal Code, 2024, article 69º-B)

Similarly, consistent housing provides the fundamental framework for change, as it builds the foundations for developing social capital and other social ties (Kras et al., 2016). However, sex offenders face increased difficulties in securing housing after release, particularly due to restrictions on returning to the area in which they previously lived and worked as a measure to protect the victim(s), which can isolate individuals from support networks and hinder their reintegration process (Brown et al., 2007; Levenson & Cotter, 2005a). They may also experience ostracism and other negative interactions with neighbors, such as threats, assaults, and harassment (Levenson & Cotter, 2005a). In Portugal, there are fewer formal housing restrictions for sex offenders compared, for instance, to the U.S. Since there is no public registry or community notification system regarding sexual offenders, most landlords are unaware of a tenant’s criminal background unless it is explicitly disclosed or revealed through a criminal record check, which is not always required.

Stable family ties and normative social connections are essential to promote successful reintegration since they are a source of emotional support and practical assistance and promote a sense of belonging, all of which are essential for the psychological well-being and stability of individuals reintegrating into society (Davis et al., 2012; Naser & Visher, 2006; Tewksbury & Connor, 2012). However, reuniting with their family can be challenging due to past experiences and unrealistic expectations (Naser & La Vigne, 2006; Naser & Visher, 2006; Tewksbury & Lees, 2006). For instance, in the U.S., where community notification policies publicize the identity of sexual offenders, family members often face significant negative repercussions, including strained relationships, harassment, and social stigma (Kras, 2019; Tewksbury & Connor, 2012). In contrast, in Portugal, the absence of such notification systems means that the identities of sexual offenders are not as widely known, which may mitigate the social repercussions experienced by offenders and their families, as well as the broader challenges of reintegration.

Indeed, several empirical studies have delved into the potential barriers faced by individuals with criminal backgrounds when attempting a successful re-entry into the community. More specifically, Lussier et al. (2011), while exploring the different needs experienced by sexual offenders during re-entry and community reintegration, concluded that these challenges are linked to the aforementioned barriers, such as finding housing, employment, and the absence of social support networks.

However, the possibility of successful reintegration might be enhanced by providing in-prison services for incarcerated individuals, focusing on both treatment and preparing individuals for community re-entry, including managing anticipated stigma and adjusting expectations (Moore et al., 2016).

Prison programs and activities are justified because they contribute to a wide range of objectives, including improved public safety, greater community cohesion, and better functioning of the offender—contributing to several beneficial outcomes, such as reduced recidivism, higher employment rates, stable housing for the offender and better family interactions (Lawrence et al., 2002). Specifically, cognitive-behavioral therapy has been considered the most effective in the treatment of sex offenders since it allows the identification of internal factors that contribute to understanding their inappropriate behavior and implementing behavioral changes to reduce risk (Lösel & Schmucker, 2005). However, according to Bullock and Bunce (2020), incarcerated individuals believed the prison system took little responsibility for monitoring and rehabilitation. Consequently, many incarcerated individuals complained about the difficulty in obtaining necessary support, characterizing the available support as limited and weak.

Moreover, interventions in prison are more likely to be effective if they comprehend a follow-up after the release (Maguire & Raynor, 2006). Therefore, parole can constitute a particularly prolific period to promote a sense of personal agency and provide access to sources of human and social capital, which can help formerly incarcerated individuals obtain the vocational, interpersonal, and personal skills they need to forge new pro-social roles that will naturally facilitate their reintegration into society (Lawrence et al., 2002). In Portugal, however, such programs within the prison system or during community supervision are often limited in scope, lacking a variety of interventions that address the diverse needs of different types of sexual offenders, ultimately reducing their potential to support a successful reintegration process (García & Pereira, 2018).

In conclusion, recognizing that community re-entry can be a significant challenge for sex offenders, the current study seeks to identify the specific challenges they encounter upon returning to the community and to explore participants’ perceptions regarding the strategies and support during the periods of incarceration and probation, as outlined below.

## Current Study

The current study adopted a qualitative nature and sought to explore and describe the perceptions and experiences of formerly incarcerated individuals convicted of a sexual offense about their community reintegration process. The following research questions guided the research:

(i) How do participants perceive and describe their process of reentering the community after serving a prison sentence concerning housing, employment, family and social relationships, and perceived stigma?
(ii) What is the perceived relevance of the strategies and support provided during the prison sentence in the preparation for release and community reintegration?
(iii) How important are the strategies and support provided during parole for the community reintegration process?

## Method

### Participants

The study was developed with a convenience sample (*n* = 10) of male former incarcerated individuals, aged between 32 and 75 years (*M* = 49; *SD* = 16.62), convicted of a sexual crime, who were on parole at the time of the data gathering. The participants were under the supervision of DGRSP’s (General Directorate of Rehabilitation and Prison Services) parole officers (mean parole duration was 3 years and 10 months; *SD* = 1.28) and had no prior criminal records. The inclusion and exclusion criteria included being male, having 18 years of age or more, not presenting mental health problems or paraphilias, and having been convicted of a sexual offense (involving sexual abuse of minors, rape, child pornography, and sexual abuse of a person incapable of resistance) and serving a prison sentence.

### Procedure

The sample was selected from the Directorate-General for Reintegration and Prison Services (DGRSP), focusing specifically on the Porto rehabilitation services. These teams operate within the central cities of the Porto metropolitan area, a region home to approximately 1.3 million inhabitants. Their primary responsibility involves supervising sexual offenders, all of whom are conditionally released, during their parole period. After being granted the authorization for data collection by the DGRSP General Director, the collaboration with parole officers allowed the identification of 12 eligible participants (considering the above-mentioned inclusion and exclusion criteria). Of the 12 identified potential participants, 10 were effectively interviewed, as one declined to participate, and another was unavailable to attend the interview due to health issues. Parole officers made the initial contact with the potential participants, provided them with a brief description of the study, and scheduled the interviews on days that aligned with participants’ follow-up appointments to minimize disruptions to their daily lives and the activities of the rehabilitation teams. The interviews were conducted in a private office provided by the rehabilitation teams, within the building where they carry out their activities.

### Interviews

This research comprised the development of semi-structured interviews (*n* = 10) with formerly incarcerated individuals convicted of a sexual crime who were on parole. The semi-structured interviews allow the respondent to provide in-depth, reflective, and personalized responses and the researcher to access the impressions, meanings, and perspectives these individuals hold regarding the world that constitutes their lived experience (Kvale, 2007; Maxfield & Babbie, 2015).

Each interview lasted approximately 1 hr and was conceived and conducted to gather information about specific topics directly related to this study’s goals and research questions. To that end, an interview guide was prepared, focusing on three major topics: the former incarcerated individual’s reintegration process (i.e., housing, employment, family, and social relationships), the institutional support and supervision (while serving the prison sentence and during parole), and the perceived stigma and discrimination. This interview guide included questions such as: “How would you describe the overall support from prison staff during your sentence and in preparation for release”; “How would you describe the support from parole services and your relationship with your parole officer?”; or “Did you experience any particular difficulties upon returning to community life?”

The interviews began by thanking the participants for their involvement in the study, followed by a thorough explanation of its purpose, objectives, and data collection procedures. Participants were also reassured about confidentiality, anonymity, and the voluntary nature of their participation.

### Analysis

The data was examined via thematic analysis. This data analysis technique is a flexible and interpretative method that identifies concepts, ideas, and meanings underlying the explicit content of the data. It also identifies patterns or themes within the data, making it suitable for addressing several research questions and analyzing different types of data (Braun & Clarke, 2013; Braun et al., 2019). The thematic analysis comprised several stages: (i) detailed transcription of the interview content; (ii) data immersion process, involving listening to the recorded content, reading and re-reading textual data, making notes on relevant aspects related to the research questions, and seeking connections and specificities; (iii) data codification, which involved a more detailed and systematic process of identifying the meaning of the data, organizing it around similar content and meanings; (iv) construction of preliminary themes, by reviewing codes and grouping data to identify similarities and overlaps; (v) reviewing and defining the themes to determine how well they fit with the coded data and the overall dataset; and (vi) the final phase involved the reporting of the results while ensuring that the constructed themes remain close to the data and address the research questions (Braun & Clarke, 2013; Braun et al., 2019).

### Ethical Reflections

The study was approved by the Ethics Committee of the Faculty of Law of the University of Porto and the Director-General of DGRSP. The research team provided the participants with oral and written information on the study (i.e., goals, data gathering, and ethical procedures), emphasizing the voluntary nature of their participation and the right to withdraw from the study at any time without explaining the reasons or having any consequences. Confidential data treatment was highlighted, and it was explained that the results were to be presented anonymously. Written, informed consent was obtained from all the participants, ensuring their authorization to audio-record the interviews.

## Findings

The following sections outline the evidence obtained through thematic analysis of the semi-structured interviews.

### Understanding the Perception of the Transitions Processes from Prison to Community

All formerly incarcerated individuals perceived returning to the community as a “difficult” (P10) or “complicated” (P5) process, some even noticing that they wouldn’t expect it to be so hard: “I didn’t expect it to be so. . .complex and, and difficult” (P4).

The interviewees gave several reasons for the complexity of this process, such as the fear of engaging in deviant behaviors again, structural and organizational changes in the community they returned to, changes in their personal situation (e.g., socioeconomic status), and the nature of the offense committed. The latter reason is said to contribute to developing feelings of shame towards the community, friends, and family after leaving prison: “Because facing society with a crime like this is complicated. (. . .) It’s difficult with your family, neighbors, and all that*”* (P10). More specifically, this latter reason resulted in constraints in interacting with people they knew due to uncertainty about how they would react to their release. Also, two interviewees described difficult experiences of stigmatization in the community setting, mostly associated with the offense committed rather than with the former incarcerated individual’s status, referring to situations of “persecution” (P4), attacks, and “threats” (P5), especially from people who were once close to them (e.g., ex-wife), and from people they didn’t know:

They even. . .made some. . .some flyers with, with my photo and inscriptions and. . .pasted them on the walls of the street where I lived. . .so, that was, it was a little, a little is being minimalist, it was a little. . .traumatizing (P4)

In response to these challenges, five participants reflected on their different strategies to cope with such situations. The most frequently mentioned strategies included isolating themselves, avoiding interaction with minors, avoiding certain places, refraining from going out alone, and avoiding people they know: “there are things I avoid, there are places I avoid going. . .I avoid going out alone” (P7) and “I’ve had lunch with my parents in a restaurant, and I’ve seen a child sit next to me and I’ve walked away” (P5).

### Social Factors Influencing Community Reintegration: The Impact of Housing, Employment, and Affective and Social Relationships

Securing a stable house is one of the most pressing tasks upon the incarcerated individual’s release. In this regard, all participants conveyed that, upon leaving prison, they did not need to search for housing since most of the participants resorted to the support of family and friends, moving in with them, or returning to their pre-imprisonment houses. Only two interviewees reported seeking assistance from social institutions to secure housing.

Regarding relationships with neighbors, almost all participants described having “good” (P9) and “friendly” (P7) relations, leading to a harmonious coexistence: “I don’t have any problems with my neighbors” (P10). In fact, the interviewees considered having stable housing and positive relationships with their neighbors important for a successful reintegration process, mitigating the need to isolate themselves, fostering a sense of comfort in their residential area, and avoiding conflicts. Nonetheless, four participants admitted to colder relationships. For example, one participant referred to avoiding interactions with neighbors due to previous experiences perceived as humiliating or stigmatizing, mainly related to the type of offense committed:

I don’t have any contact, I even avoid (. . .) because it’s like this, I’ve had situations of someone coming up to me “paedophile” (. . .) and so. . .I don’t go, I don’t, I don’t talk to, to anyone (P5)

Employment was also explored as a major contributing factor for a successful reintegration. During the interviews, five interviewees reported being unemployed, with one participant engaging in an internship at an employment and vocational training institute dedicated to recently released incarcerated individuals. Only two participants have returned to the positions they held before their imprisonment. Then, three interviewees were in the process of retiring, and thus, this dimension was not largely discussed with them. Nevertheless, one of these participants emphasized the challenges of seeking employment after leaving prison for individuals convicted of a sexual offense, sharing his belief that unemployment is a contributing factor to recidivism. Four other participants also mentioned that their criminal record could be an obstacle, while others have experienced long periods of waiting for a response.

When asked about the importance of employment for the community reintegration process, the interviewees emphasized its significance for two main reasons: it is essential for achieving greater financial independence and for occupying time and providing a distraction:

I can’t always depend on my family. . . they help, but I can’t, I can’t always depend on them (P8)

The more - the more stable life is. . ., the less a person tends to look for distractions in other things (P7)

Lastly, affective and social relationships, namely with intimate partners, family members, and friends, were also discussed in the current research.

When it comes to intimate relationships, six participants who were involved in such partnerships share positive experiences marked by a strong foundation of friendship and companionship. Additionally, they expressed encountering no challenges in sustaining or developing connections of this kind. They emphasized the importance of these relationships for successful community reintegration, mainly due to the support, joy, and affection they feel from their partners. However, they also recognized certain detrimental consequences and changes in their partners associated with their conviction, including in their physical, emotional, and psychological health:

She [wife] never abandoned me (. . .) since I went to prison, she went through a lot with me (. . .), and because if I, if I had been there, she wouldn’t have collapsed, she wouldn’t have had this problem [referring to the strokes] (P10)

Regarding relationships with other family members, seven participants described them as “impeccable” (P8), normal and unchanged, particularly with parents and siblings. The family was portrayed as a crucial source of support, especially during imprisonment at both financial and emotional levels, and a source of control and supervision: “When I should have been taking care of them, they were the ones taking care of me. . .my parents always supported me” (P5). However, one participant perceived these relationships as simultaneously important and painful since “they will never forget what happened” (P4).

Finally, regarding social relationships, the participants referred to having “good” friends (P8). Only two participants refrained from forming friendships due to concerns about negative influences, with one expressing, “they are bad company, it’s more like drugs and consumption and. . .. I prefer to stay away” (P6). While four participants reported changes in friendships, mentioning distancing and increased supervision from friends, two others felt their friendships remained unchanged. Regarding difficulties in maintaining or developing these relationships, some participants expressed fear of their friends’ reactions, while others were open to forming new connections.

### Support and Supervision During Prison and Parole: Divergent Perceptions on Its Importance to a Successful Community Reintegration

One of the aspects systematically addressed in the interviews was the support provided during the prison sentence. The prevailing perception among the interviewees was that there were some deficiencies in the assistance provided to them during incarceration, and it was often deemed as insufficient to address specific needs, including personal, familiar, and psychological, with some interviewees even expressing the need to seek this assistance elsewhere. Furthermore, concerning preparation for release, six participants noted the absence of specialized support to facilitate their transition from prison life to the community, as follows:

I was waiting a year, two years for someone to go to the psychiatrist, to go to something. . . and we needed to be attended to (. . .) There were many times when we needed it, we asked to be accompanied, and they weren’t. . . they weren’t interested (P8)

Although prevalent, this perception was not unanimous. In fact, four participants reported receiving adequate support during their time in prison, which they described as “good” (P6). For example, two participants mentioned the right to prison furlough, while another participant acknowledged the help provided by the prison staff in addressing financial and accommodation needs after release.

Besides the assistance provided, only three participants reported having had the opportunity to attend program integration (addressing sexual issues) and other related activities while in prison. Those who participated in such programs viewed them as somewhat beneficial in terms of taking responsibility for the crime committed and internalizing the value of the conduct:

We were able to. . .open up and get our doubts out of our heads because. . .when we go in, we’re going to say that we didn’t do it wrong. . .either because the victims wanted it or because. . .lots of situations, but we did it wrong. And then there’s the part where we start to understand why. . .we realize that we’re the ones who made mistakes (P5).

In relation to parole time, six participants described the support they received while on parole as “good” (P8), “nice” (P9), or “beneficial” (P4), with two participants mentioning that they felt they received “a lot more” (P8) support during parole than when they were in prison. However, the other four interviewees expressed a less favorable perception, referring to the lack of support from social reintegration services or its insufficiency.

Despite these different perspectives on the quantity and quality of the support provided, participants talked about the importance and contribution of the support they received, specifically regarding their adaptation and reintegration into the community. Four participants considered it important, emphasizing its significance in preventing recidivism, acquiring the necessary skills to conduct themselves daily or in a potentially conflicting situation, developing knowledge about the victims and their needs, and, finally, reaffirming the individual’s commitment to society while in freedom. Three other participants did not express such positive views, referring to parole support as indifferent, insufficient, limiting and perceiving the periodic presentations as a “massacre” (P5) that revives painful memories:

I had two months there that if I didn’t have parents. . .I wouldn’t know. . .I’d be walking around. . .I think the counseling fails. . ..no, I think our system fails a lot in this respect (P7).

Five participants also referred to attending programs addressing sexual issues during their parole. They considered these appointments essential for discussing their crime and past experiences, perceiving them as crucial for preventing recidivism and fostering personal openness and self-knowledge: “It can have some benefit, in the sense of not returning to a life of crime (. . .) in the sense of. . .getting things off my chest, talking about things that I never thought I could talk about” (P2). While the majority felt that the counseling provided significant support, it is important to note that not all participants shared this viewpoint. In fact, one of the participants believed that these appointments did not bring anything new or help him in any way.

## Discussion

The current study explored sex offender’s perceptions and experiences concerning their community reintegration process after completing a prison sentence.

More specifically, this study sought to explore key aspects for a successful reintegration, such as housing, employment, and family and social relationships. Most participants reported no difficulties securing housing, as they relied on their family and friends’ support or returned to their pre-incarceration homes. This contrasts with previous studies highlighting the challenges sexual offenders face in accessing housing, particularly in countries with public notification systems and housing restrictions (Levenson & Cotter, 2005a; Liem & Weggemans, 2018; Tewksbury & Copes, 2012). In Portugal, the absence of such systems might have contributed to this finding, as most landlords and neighbors are unaware of the offenders’ past, reducing the social stigma and legal barriers typically encountered. This less punitive and more rehabilitative approach could explain the softer reintegration process observed in this study, with participants benefiting from strong family or community support. Nevertheless, this aspect comes as very positive as consistent housing provides the fundamental structure for change, building the foundation upon which social capital and other social ties can be developed (Kras et al., 2016).

In terms of employment, most interviewees reported being unemployed, with only two being able to return to their previous occupations. These findings align with previous studies highlighting several difficulties most sexual offenders face when seeking employment after incarceration, namely the nature of their crime, extended periods of incarceration, low education, limited work experience, and employer bias (Brown et al., 2007; Liem & Weggemans, 2018; Rydberg, 2018). However, the difficulties in securing employment faced by the participants are not primarily due to legal restrictions or occupational bans, but rather to broader societal challenges that affect many individuals, both ex-offenders and the general population. While there are some limitations for sex offenders, particularly concerning working in public service jobs or positions involving direct contact with vulnerable populations (e.g., children), a criminal record is not typically requested for most employment positions. Nonetheless, employment remains a crucial factor for successful reintegration, providing financial stability, a sense of purpose, and an opportunity to develop pro-social skills, all of which contribute to reducing the risk of recidivism (Brown et al., 2007; Davis et al., 2012).

As previously mentioned, the individuals’ relationship network is another important element in the reintegration process. Contrary to what was expected, in this study, the participants reported relatively few difficulties maintaining family and social relationships post-release, despite the stigma typically associated with sexual offenses (Liem & Weggemans, 2018; Tewksbury & Connor, 2012; Tewksbury & Copes, 2012). Once again, the absence of public exposure means that, in most cases, family members and friends are unaware of the offender’s criminal history—and, as a result, may not face the same societal pressure to distance themselves, and offenders themselves are less likely to experience public shaming or harassment, therefore contributing to stronger family ties and more positive social reintegration outcomes. Another aspect that may have also contributed to this result is attributed to the timing of interviews, often conducted shortly after release, possibly during a “honeymoon” or family reunification period. However, it is noted that as re-entry pressures persist over time, the support and quality of family relationships may decrease (Naser & La Vigne, 2006). Therefore, follow-up interviews with participants would be valuable to explore changes throughout the parole period. Additionally, while some participants expressed some concerns over increased supervision or distancing from friends, these relationships generally appear to have remained intact.

The current study also sought to understand the perceived relevance of the support provided during the prison sentence in preparation for release and community reintegration. A significant number of participants expressed dissatisfaction with the lack of preparation for re-entry, emphasizing the absence of programs tailored to their criminogenic needs. In Portugal, where the legal and institutional framework often prioritizes psychological support within the prison system, these interventions are not only limited but also voluntary and typically not mandated by the courts. As a result, many offenders do not participate in these programs due to lack of motivation, limited availability, and insufficient focus on the specific issues related to sexual offenses, particularly those committed against adults (Gomes et al., 2018). The most common programs available tend to focus on sexual offenses involving children, leaving a gap in treatment for those who have committed offenses against adults. Moreover, even when programs are available, they remain insufficient to meet the needs of the large number of incarcerated individuals returning to the community, further exacerbating the challenges faced during reintegration (Bullock & Bunce, 2020; Lussier et al., 2011). Furthermore, in prison, individuals may access clinical services, including psychologists and psychiatrists, and receive some support from re-education officers. However, participants did not mention receiving psychological support in prison, describing re-education officers as handling mainly administrative or practical matters. Psychological support was only referenced post-release, particularly under community supervision. These limitations reinforce the need for a more structured and integrated approach to re-entry preparation that addresses criminogenic needs and promotes problem-solving skills, social interaction, and attitude changes to reduce offending behavior (Muntingh, 2009).

Another aim of this research was to explore the perceptions of support during parole. Participants revealed different notions of the monitoring quality by reintegration services. While some interviewees considered it adequate, having a positive notion of the accompaniment they were subjected to, others felt neglected, describing it as brief visits or mere follow-up interviews. In Portugal, the approach to post-release supervision tends to be less restrictive compared to other countries. The support provided is primarily focused on social aspects, such as checking if the individual is employed, monitoring their family situation, assisting with physical or mental health issues, and offering psychological support (Gomes et al., 2018). There is, however, a notable absence of specialized treatment for sexual offenders. The absence of specialized treatment targeting dynamic risk factors has been defined as an important constraint during community reintegration (Lussier et al., 2011). This deficiency could negatively impact the efforts to prevent recidivism, as treatment is crucial in fostering individual responsibility and disrupting the offending cycle (Jeglic et al., 2011). Although parole is conceived to encourage reflection on change and to empower skill-building (King, 2013), the absence of specialized programs for sexual offenders in Portugal limits the effectiveness of this period in facilitating personal change and long-term reintegration.

Lastly, the cultural and legal differences between Portugal and other countries, such as the U.S., might have influenced the findings about the social reintegration of sexual offenders, given the marked differences in legislation and societal perceptions in each country. For example, in the U.S., laws related to sexual offenders are extremely stringent, with housing restrictions, constant monitoring, and public sex offender registries, which often perpetuate the stigma and make reintegration more challenging. These laws impose restrictions on access to certain areas, such as schools or parks, and enforce permanent registration obligations, which may contribute to a cycle of social marginalization and increased difficulty regarding social reintegration (Levenson & Cotter, 2005a). This legal reality contrasts sharply with the Portuguese one, where legal and social restrictions can be perceived as more lenient and less punitive. In fact, there are no public registries of sexual offenders, and the laws do not impose such severe limitations on housing or employment, allowing individuals more opportunities to rebuild their lives with less state interference. These legal and cultural factors can directly influence the challenges these individuals face during reintegration, from societal barriers to the availability of support systems aimed at reducing recidivism (Brown et al., 2007).

### Limitations and Future Research

Despite its contributions, this study has notable limitations. Firstly, it did not track participants’ full process of incarceration, transition to community, or the process of community reintegration, which hinders a comprehensive understanding. Future research should address these aspects to provide a fuller picture of re-entry. Furthermore, some interviews took place immediately after participants re-entered the community, which might have limited their ability to reflect on their experiences. Follow-up interviews could offer more comprehensive insights. Additionally, the sample size, while valuable, may not have reached full theoretical saturation, suggesting that larger samples are needed in future studies. Finally, social desirability bias presents another limitation, as participants might have presented themselves more favorably, especially under parole supervision. Future studies should consider methods to minimize this bias and enhance data accuracy.

Despite the outlined limitations, this study contributes to understanding the community reintegration of sexual offenders post-release, an area with limited research. Its qualitative insights can help shape more effective public protection policies and practices, as well as guide justice systems and prisons in improving re-entry strategies.

## Conclusion

Successful reintegration of sex offenders hinges on key social factors such as stable housing, family support, community networks, and employment (Kras et al., 2016). This study emphasizes the importance of strengthening support during the transition from prison to community, particularly in addressing stigma-related issues and assisting with employment (Brown et al., 2007; Muntingh, 2009). Additionally, treatment programs like cognitive-behavioral therapy during incarceration and parole, combined with ongoing support, can help offenders adjust, promoting a holistic approach that addresses practical, psychological, and social needs for successful re-entry (Brown et al., 2007; Lösel & Schmucker, 2005; Maguire & Raynor, 2006).

## References

[bibr1-0306624X251324661] Adam Walsh Child Protection and Safety Act. (2006). Pub. L. No. 109-248, 120 Stat. 587. https://www.congress.gov/bill/109th-congress/house-bill/4472

[bibr2-0306624X251324661] Braun V. Clarke V. (2013). Successful qualitative research: A practical guide for beginners. Sage.

[bibr3-0306624X251324661] Braun V. Clarke V. Hayfield N. Terry G. (2019). Thematic analysis. In Liamputtong P. (Ed.), Handbook of research methods in health social sciences (pp. 843–860). Springer.

[bibr4-0306624X251324661] Brown K. Spencer J. Deakin J. (2007). The reintegration of sex offenders: Barriers and opportunities for employment. The Howard Journal of Criminal Justice, 46(1), 32–42.

[bibr5-0306624X251324661] Bullock K. Bunce A. (2020). ‘The prison don’t talk to you about getting out of prison’: On why prisons in England and Wales fail to rehabilitate prisoners. Criminology & Criminal Justice, 20(1), 111–127.

[bibr6-0306624X251324661] Burchfield K. B. Mingus W. (2008). Not in my neighborhood: Assessing registered sex offenders’ experiences with local social capital and social control. Criminal Justice and Behavior, 35(3), 356–374.

[bibr7-0306624X251324661] Davis C. Bahr S. J. Ward C. (2012). The process of offender reintegration: Perceptions of what helps prisoners reenter society. Criminology & Criminal Justice, 13(4), 446–469.

[bibr8-0306624X251324661] Diário da República. (2015). Law No. 103/2015, of August 24. Diário da República No. 164/2015, Series I of August 24, 2015. https://diariodarepublica.pt/dr/detalhe/lei/103-2015-70086390

[bibr9-0306624X251324661] García G. S. J. Pereira S. A. (2018). Perceções dos reclusos sobre a vida na prisão e o processo de ressocialização. Psique, 14, 8–29.

[bibr10-0306624X251324661] Gomes S. Leote De Carvalho M. J. Oliveira R. V. Duarte V. (2018). Trends and challenges in the Portuguese penitentiary system: From law to practice. Antigone, 13(1/2), 61–102.

[bibr11-0306624X251324661] Harris A. J. Socia K. M. (2016). What’s in a name? Evaluating the effects of the “sex offender” label on public opinions and beliefs. Sexual Abuse, 28(7), 660–678. 25542837 10.1177/1079063214564391

[bibr12-0306624X251324661] Jeglic E. L. Maile C. Calkins-Mercado C. (2011). Treatment of offender populations: Implications for risk management and community reintegration. In Gideon L. Sung H. (Eds.), Rethinking corrections. Rehabilitation, reentry, and reintegration (pp. 37–70). Sage.

[bibr13-0306624X251324661] King S. (2013). Assisted desistance and experiences of probation supervision. Probation Journal, 60(2), 136–151.

[bibr14-0306624X251324661] Kras K. R. (2019). Can social support overcome the individual and structural challenges of being a sex offender? Assessing the social support-recidivism link. International Journal of Offender Therapy and Comparative Criminology, 63(1), 32–54. 29947562 10.1177/0306624X18784191

[bibr15-0306624X251324661] Kras K. R. Pleggenkuhle B. Huebner B. M. (2016). A new way of doing time on the outside: Sex offenders’ pathways in and out of a transitional housing facility. International Journal of Offender Therapy and Comparative Criminology, 60(5), 512–534. 25326464 10.1177/0306624X14554194

[bibr16-0306624X251324661] Kvale S. (2007). Doing interviews. Sage Publications.

[bibr17-0306624X251324661] Lawrence S. Mears D. P. Dubin G. Travis J. (2002, May). The practice and promise of prison programming. Report, The Urban Institute.

[bibr18-0306624X251324661] Levenson J. S. Cotter L. P. (2005a). The impact of sex offender residence restrictions: 1,000 feet from danger or one step from absurd? International Journal of Offender Therapy and Comparative Criminology, 49(2), 168–178. 15746268 10.1177/0306624X04271304

[bibr19-0306624X251324661] Levenson J. S. Cotter L. P. (2005b). The effect of Megan’s Law on sex offender reintegration. Journal of Contemporary Criminal Justice, 21(1), 49–66.

[bibr20-0306624X251324661] Liem M. Weggemans D. (2018). Reintegration among high-profile ex-offenders. Journal of Developmental and Life-Course Criminology, 4(4), 473–490. 30873339 10.1007/s40865-018-0093-x PMC6390714

[bibr21-0306624X251324661] Lösel F. Schmucker M. (2005). The effectiveness of treatment for sexual offenders: A comprehensive meta-analysis. Journal of Experimental Criminology, 1, 117–146.

[bibr22-0306624X251324661] Lussier P. Dahabieh M. Deslauriers-Varin N. Thomson C. (2011). Community reintegration of violent and sexual offenders. Issues and challenges for community risk management. In Gideon L. Sung H. (Eds.), Rethinking corrections. Rehabilitation, reentry, and reintegration (pp. 219–252). Sage.

[bibr23-0306624X251324661] Maguire M. Raynor P. (2006). How the resettlement of prisoners promotes desistance from crime: Or does it? Criminology and Criminal Justice, 6(1), 19–38.

[bibr24-0306624X251324661] Maxfield M. G. Babbie E. R. (2015). Research methods for criminal justice and criminology. Cengage Learning.

[bibr25-0306624X251324661] McAlinden A. M. (2006). Managing risk: From regulation to the reintegration of sexual offenders. Criminology & Criminal Justice, 6(2), 197–218.

[bibr26-0306624X251324661] Mingus W. Burchfield K. B. (2012). From prison to integration: Applying modified labeling theory to sex offenders. Criminal Justice Studies, 25(1), 97–109.

[bibr27-0306624X251324661] Moore K. E. Stuewig J. B. Tangney J. P. (2016). The effect of stigma on criminal offenders’ functioning: A longitudinal mediational model. Deviant Behavior, 37(2), 196–218. 26973364 10.1080/01639625.2014.1004035 PMC4788463

[bibr28-0306624X251324661] Muntingh L. (2009). Ex-prisoners’ views on imprisonment and re-entry. CSPRI – Community Law Centre.

[bibr29-0306624X251324661] Naser R. L. La Vigne N. G. (2006). Family support in the prisoner reentry process: Expectations and realities. Journal of Offender Rehabilitation, 43(1), 93–106.

[bibr30-0306624X251324661] Naser R. L. Visher C. A. (2006). Family members’ experiences with incarceration and reentry. Western Criminology Review, 7(2), 20–31.

[bibr31-0306624X251324661] Portuguese Penal Code. (2024). Diário da República No. 63/1995, Series I-A of March 15, 1995. https://diariodarepublica.pt/dr/legislacao-consolidada/decreto-lei/1995-34437675

[bibr32-0306624X251324661] Robbers M. L. (2009). Lifers on the outside: Sex offenders and disintegrative shaming. International Journal of Offender Therapy and Comparative Criminology, 53(1), 5–28. 18268079 10.1177/0306624X07312953

[bibr33-0306624X251324661] Rydberg J. (2018). Employment and housing challenges experienced by sex offenders during reentry on parole. Corrections: Policy, Practice and Research, 3(1), 15–37.

[bibr34-0306624X251324661] Schiavone S. K. Jeglic E. L. (2009). Public perception of sex offender social policies and the impact on sex offenders. International Journal of Offender Therapy and Comparative Criminology, 53(6), 679–695. 18728128 10.1177/0306624X08323454

[bibr35-0306624X251324661] Solomon A. L. Johnson K. D. Travis J. McBride E. C. (2004). From prison to work: The employment dimensions of prisoner reentry. A report of the reentry roundtable (pp. 1–32). Urban Institute.

[bibr36-0306624X251324661] Stoll M. A. Bushway S. D. (2008). The effect of criminal background checks on hiring ex-offenders. Criminology & Public Policy, 7(3), 371–404.

[bibr37-0306624X251324661] Tewksbury R. Connor D. P. (2012). Incarcerated sex offenders’ perceptions of family relationships: Previous experiences and future expectations. Western Criminology Review, 13(2), 25–35.

[bibr38-0306624X251324661] Tewksbury R. Copes H. (2012). Incarcerated sex offenders’ expectations for reentry. The Prison Journal, 93(1), 102–122.

[bibr39-0306624X251324661] Tewksbury R. Lees M. (2006). Perceptions of sex offender registration: Collateral consequences and community experiences. Sociological Spectrum, 26(3), 309–334.

[bibr40-0306624X251324661] Visher C. A. Travis J. (2003). Transitions from prison to community: Understanding individual pathways. Annual Review of Sociology, 29, 89–113.

